# Membrane lipid adaptation of soil *Bacteroidetes* isolates to temperature and pH

**DOI:** 10.3389/fmicb.2023.1032032

**Published:** 2023-03-06

**Authors:** Eve Hellequin, Sylvie Collin, Marina Seder-Colomina, Pierre Véquaud, Christelle Anquetil, Adrienne Kish, Arnaud Huguet

**Affiliations:** ^1^Sorbonne Université, CNRS, EPHE, PSL, UMR METIS, Paris, France; ^2^Muséum National d'Histoire naturelle, CNRS, Unité Molécules de Communication et Adaptation des Microorganismes UMR7245 MCAM, Paris, France

**Keywords:** 3-hydroxy fatty acids, gram-negative bacteria, soil, temperature, pH, adaptation, proxy, *Bacteroidetes*

## Abstract

3-hydroxy fatty acids (3-OH FAs) are characteristic components of the Gram-negative bacterial membrane, recently proposed as promising temperature and pH (paleo) proxies in soil. Nevertheless, to date, the relationships between the 3-OH FA distribution and temperature/pH are only based on empirical studies, with no ground truthing work at the microbial level. This work investigated the influence of growth temperature and pH on the lipid composition of three strains of soil Gram-negative bacteria belonging to the *Bacteroidetes* phylum. Even though non-hydroxy FAs were more abundant than 3-OH FAs in the investigated strains, our results suggest that 3-OH FAs are involved in the membrane adaptation of these bacteria to temperature. The strains shared a common adaptation mechanism to temperature, with a significant increase in the ratio of *anteiso* vs. *iso* or *normal* 3-OH FAs at lower temperature. In contrast with temperature, no common adaptation mechanism to pH was observed, as the variations in the FA lipid profiles differed from one strain to another. We suggest that models reconstructing environmental changes in soils should include the whole suite of 3-OH FAs present in the membrane of Gram-negative bacteria, as all of them could be influenced by temperature or pH at the microbial level.

## 1. Introduction

Microorganisms (bacteria, archaea and some eukaryotes) are able to adjust their membrane lipid composition in response to the prevailing environmental conditions in order to ensure the optimal state of the cellular membrane by maintaining the appropriate fluidity (Hazel and Williams, 1990; Denich et al., 2003; Mykytczuk et al., 2007). Such a strategy is termed “homeoviscous adaptation” (Sinensky, 1974). To regulate membrane fluidity in response to varying environmental conditions bacteria can change the number of unsaturations, positions of ramifications or chain length of the carbon skeleton in their membrane lipids — mainly fatty acids — (Reizer et al., 1985; Prado et al., 1988; Suutari and Laakso, 1992). Other mechanisms for membrane adaptation to temperature or pH changes can include hydroxylation of intact polar lipids (Moore et al., 2015) or synthesis of carotenoids in psychrophilic species (Chattopadhyay et al., 1997; Jagannadham et al., 2000; Gruszecki and Strzałka, 2005).

In the same way, the structure of glycerol dialkyl glycerol tetraethers (GDGTs), which are membrane lipids biosynthesized by archaea and some bacteria, is known to be related to environmental parameters (Schouten et al., 2013). In aquatic environments, the relative abundance of different isoprenoid GDGTs produced by members of the archaeal phylum *Thaumarchaeota* was correlated with the water surface temperature, leading to the development of a temperature proxy (Schouten et al., 2002), the TEX_86_ index, largely applied to marine and lacustrine paleorecords (Castañeda et al., 2010; Berke et al., 2012). Another type of GDGTs with branched alkyl chains – so-called branched GDGTs (brGDGTs) – were suggested to be produced by bacteria and are ubiquitous in aquatic and terrestrial environments (Schouten et al., 2013). Their analysis in soils/peats (Véquaud et al., 2022) and lake sediments (Martínez-Sosa et al., 2021) distributed worldwide showed that their structure varies mainly with air temperature and soil pH, making them increasingly used as temperature and pH paleoproxies. BrGDGTs are the only microbial organic proxies which can be used for temperature reconstructions in both aquatic and terrestrial settings. Nevertheless, paleoenvironmental data derived from these molecules have to be interpreted with care, as (i) their source microorganisms remain unknown, although some might belong to the phylum *Acidobacteria* (Sinninghe Damsteé et al., 2011; Sinninghe Damsté et al., 2018) and (ii) high uncertainty is associated with global brGDGT-mean annual air temperature (MAAT) calibrations (>4°C; Véquaud et al., 2022). The development of new environmental proxies, independent and complementary to brGDGTs, is crucial to improve the reliability and accuracy of continental reconstructions.

Recently, Wang et al. (2016) suggested that other bacterial lipids, so called 3-hydroxy fatty acids (3-OH FAs), could be used as such a proxy. These compounds, containing 10–18 carbon atoms and a hydroxyl group in third position, are characteristic components of the Gram-negative bacterial membrane (e.g., Szponar et al., 2002, 2003; Keinänen et al., 2003). The membrane component of the cell wall of Gram-negative bacteria is more complex than that of Gram-positive cells, since it is composed of two distinct membranes, the outer membrane and the inner membrane, enclosing a thin space, the periplasm. The inner leaflet of the outer membrane is generally thought to be composed of phospholipids, but recent data clearly state that lipids other than phospholipids can be found in the outer membrane (Sohlenkamp and Geiger, 2016 and references therein). Aminoacid lipids such as ornithine lipids have been identified in members of the *Cytophaga*, *Bacteroidetes*, *Flavobacterium* groups as well as in *Proteobacteria* (Sohlenkamp and Geiger, 2016). An example is *Flavobacterium johnsoniae* whose very unusual membrane lipid composition includes only one phospholipid (phosphatidylethanolamine) among the major membrane-forming lipids, its membranes being also composed of sulfonolipids, ornithine lipids, glycine lipids and serineglycine lipids (Vences-Guzmán et al., 2021). Ornithine lipids contain two fatty acyl groups, with a 3-hydroxy fatty acyl group attached to the α-amino group of ornithine (Vences-Guzmán et al., 2012). Very little data are available on the exact localization of these lipids, whether in the inner or the outer leaflet of the outer membrane. The outer leaflet of the outer membrane is thought to be mainly composed of lipopolysaccharide (LPS; Kamio and Nikaido, 1976). The LPS has a tripartite structure with the hydrophobic lipid A, the core oligosaccharide and the O-antigen polysaccharide, the two latter being hydrophilic. Lipid A, mainly constituted of 3-OH FAs (Raetz et al., 2007), anchors the LPS to the outer membrane and acts as a signaling molecule for the innate immune system. Three types of 3-OH FAs can be distinguished according to the presence and position of a methyl group on the carbon chain: *normal* (i.e., straight), *iso* (methyl in penultimate position) or *anteiso* (methyl group in the antepenultimate position).

Gram-negative bacteria are ubiquitous in aquatic and terrestrial environments. 3-OH FAs have been previously used to detect and quantify Gram-negative communities in various types of samples originating from terrestrial, aquatic and atmospheric environments (Wakeham et al., 2003; Lee et al., 2004; Tyagi et al., 2015, 2016). Nevertheless, until recently, these lipids have been largely overlooked for paleoclimate applications. Only limited information is available regarding the response of Gram-negative bacteria and associated 3-OH FA membrane lipids to the variations of environmental parameters.

Significant local correlations were recently obtained between the relative abundance of 3-OH FAs and temperature/pH in 26 soils collected along Mt. Shennongjia (China; Wang et al., 2016). Thus, the ratio of the summed *iso* and *anteiso* to the total amount of *normal* 3-OH FAs (the so-called RIAN index) was shown to vary with soil pH (Wang et al., 2016). In addition, the *anteiso* to *normal* 3-OH FA ratios of the C_15_ and C_17_ compounds (RAN_15_ and RAN_17_ indices, respectively) were negatively and linearly correlated with MAAT. The application of 3-OH FA based-proxies to a Holocene Chinese stalagmite reinforced the potential of these compounds as terrestrial paleoenvironmental proxies (Wang et al., 2018). Very recently, the applicability of 3-OH FAs as temperature and pH proxies was further investigated at the global level using extended soil datasets with samples from all over the world (Wang et al., 2021; Véquaud et al., 2021a). Significant relationships between the relative abundance of 3-OH FAs and temperature/pH at the global scale could only be obtained using non-linear models based on machine-learning algorithms (Wang et al., 2021; Véquaud et al., 2021a), highlighting the complexity of the response of 3-OH membrane lipids to changes in environmental parameters. Such a complexity may be related to the fact that (i) the diversity of Gram-negative bacteria varies from one soil to another (Margesin et al., 2009; Siles and Margesin, 2016) and that (ii) the 3-OH FA distribution differs between Gram-negative genera and species (Oyaizu and Komagata, 1983; Goossens et al., 1986; Hedrick et al., 2009; Wang et al., 2018). Therefore, the applicability of 3-OH FAs as environmental proxies in soils could be strongly influenced by the Gram-negative bacterial diversity, as the response to temperature and pH changes may vary between species. Nevertheless, to the best of our knowledge, no work to date has been performed at the microbial level to assess the impact of environmental parameters on the 3-OH FA profiles of various Gram-negative bacterial strains from soils.

In this study, we examined for the first time the influence of temperature and pH on three different strains of soil Gram-negative bacteria belonging to the same phylum (*Bacteroidetes*) in order to better understand the effect of variable environmental parameters on the membrane lipid profile, with a focus on 3-OH-FAs and their applicability as temperature and pH proxies. We hypothesized that (i) different temperature and pH culture conditions could lead to changes in 3-OH FA distribution and that (ii) the variations in 3-OH FA lipid profile in response to temperature and pH variations could differ from one strain to another.

## 2. Materials and methods

### 2.1. Strain isolation

#### 2.1.1. Isolation of gram-negative bacteria from surface soils

Gram-negative bacterial strains were isolated from surface soils (0–10 cm depth) collected in October 2017 along a well-documented composite altitudinal transect (234–2,748 m) representative of the variations in temperature, soil characteristics and plant communities in the French Alps (Véquaud et al., 2021b). Isolation of Gram-negative bacterial strains was carried out from four contrasting soils collected in lowland, subalpine, mountainous and alpine land sites (samples 4, 19, 39, and 44, respectively, in Véquaud et al. (2021b)). The physicochemical characteristics of these soil samples are detailed in Véquaud et al. (2021b).

Bacterial strains were isolated by resuspending 2 g of each soil in 200 mL of sterile 0.9% (w/v) NaCl, incubated at 225 rpm on a rotary shaker for 20 min then left to sediment at room temperature for 40 min. The supernatant was diluted to 10^−3^ and 10^−4^ in sterile NaCl 0.9% before plating on R2A agar. Bacterial isolation was carried out at pH 7.2 or pH 5.0 (representative of the pH measured in the French Alps; Véquaud et al., 2021b) on R2A agar plates supplemented with vancomycin 10 mg/L, teicoplanin 10 mg/L, daptomycine 10 mg/L and cycloheximide 400 mg/L. For each soil, growth was monitored on eight plates at pH 7.2 and eight plates at pH 5.0, incubated at 5°C or at 25°C until no new colonies appeared. Twenty different clones covering the visible morphological differences of the colonies (colony size, shape, color, smoothness, general aspect), observed in each condition were re-isolated twice on the same medium. A single colony of each clone was then used to inoculate 10 mL of LB medium 0.1X, grown overnight at 25°C or for 48 h at 5°C before storage of the cell pellet at −80°C after resuspension in 60% glycerol, 7.5 mM MgSO_4_. Three hundred and twenty cultivable species of Gram-negative bacteria were isolated from the four aforementioned soils (Véquaud et al., 2021b) and constituted the library for our study.

#### 2.1.2. Taxonomic identification

For each bacterial clone, a single colony was used for PCR amplification of rDNA sequences. Amplification was carried out with primers 27F and 1391R (Lane, 1991) using GoTaq^®^ DNA polymerase (Promega) according to the manufacturer’s instructions, i.e., 95°C for 2 min, followed by 94°C for 30s, 60°C for 30 s, 72°C for 1min40s (35 cycles), and 72°C for 5 min. The PCR fragments were directly sequenced (Eurofins Genomics) using primer T7. A preliminary assignation was made using Standard Nucleotide Blast (Altschul et al., 1990). *Pseudomonas* generated a total of 193 first hits. The next most abundant genera were *Flavobacterium* (26 hits), *Colimonas* (17 hits), *Mucilaginibacter* (12 hits) and *Pedobacter* (9 hits). Three strains were selected from the library for further study, identified as belonging to the *Flavobacterium*, *Mucilaginibacter* and *Pedobacter* genera in the *Bacteroidetes* phylum. Further taxonomic affiliation was carried out on these clones after PCR amplification of the rDNA genes using the same primers as above and *Pfu* DNA polymerase (Promega), following the manufacturer’s instructions. The PCR fragments were cleaned, A-tailed and cloned into the pGEM-T vector. The identification of each strain was based on the sequencing of two positive plasmids using T7 and SP6 primers (Eurofins Genomics). The entire sequence was used for affiliation using EZBioCloud, database version 2021.07.07. The selected clones belonged to the *Bacteroidetes* phylum, as the C_15_ and C_17_ 3-OH FA homologues used in the RAN_15_ and RAN_17_ temperature proxies defined by Wang et al. (2016) were shown to be abundant in these bacteria (*cf.* section 4.1 for discussion).

The first strain, isolated from a calcosol collected at 232 m altitude (MAAT = 12.5°C; pH = 7.3; soil 44 in Véquaud et al., 2021b), was most closely related to *Flavobacterium pectinovorum* (98.78% similarity; GenBank accession no. OP204417). The second strain, isolated from a brunisol collected at 932 m altitude (MAAT = 8.6°C; pH = 7.2; soil 39 in Véquaud et al., 2021b), was most closely related to *Pedobacter lusitanus* NL19 (98.54% similarity; GenBank accession no. OP204418). The third strain, isolated from a rendisol collected at 2695 m altitude (MAAT = 0.4°C; pH = 6.5; soil 4 in Véquaud et al., 2021b), was most closely related to phylotype LGEL_s strain 048, *Pedobacter panaciterrae* (99.27% similarity; GenBank accession no. OP204416). In the rest of this paper, the three aforementioned strains will be referred to as strains A–C, respectively.

### 2.2. Cultivation of the strains

#### 2.2.1. Identification of the optimal growth conditions

For each strain, the optimal growth temperature at the intrinsic pH of the R2A medium (pH 7.2) was first identified. Triplicate cultures were performed for each temperature tested (5°C, 10°C, 15°C, 20°C, 25°C, and 30°C). For each culture, a colony isolated on R2A agar medium was incubated in 7 ml of sterile R2A medium at 180 rpm (Infors HT Celltron) at the temperature to be tested to set up a subculture. After a few hours or a few days (depending on the strain and the tested temperature), the optical density (OD) of the subculture was measured at 600 nm (Spectrostarnano, BMG LabTech). One hundred ml of sterile R2A medium were inoculated with a volume of the subculture, chosen to reach an OD value between 0.05 and 0.10. The growth kinetics of each strain was then established. Cultures were shaken at 180 rpm under the desired conditions in a thermostatically controlled cabinet (Pol-Eko Aparatura^®^). OD was measured every hour until the stationary phase of bacterial growth was reached. The optimum pH (5, 6, 7, or 8) was defined under the same conditions, but with the temperature set at the defined optimal growth temperature (25°C, see below). The pH of the R2A medium was adjusted with a pH meter using 1 M NaOH (pH above 7.2) or 1 M HCl (pH below 7.2). For each culture, bacterial growth curves were generated and the generation time was calculated in order to identify the optimal growth temperature and pH. 25°C was identified as the optimal growth temperature for all the strains. At 25°C, the optimal growth pH was 8 for strain A and 5 for strains B and C.

#### 2.2.2. Cultivation of the strains under different temperature and pH conditions

Once the optimum temperature and pH were identified, the strains were cultivated in triplicate in order to obtain a reference bacterial growth curve for each condition tested. The strains were cultivated at 180 rpm, either at fixed temperature (optimal growth temperature) and different pH (5, 6, 7, or 8) or at fixed pH (optimal growth pH) and different temperatures (5, 10, 15, 20, and 25°C). These reference curves were used for regular measurements of OD, in order to determine the time until stationary phase is reached. Growth phase has been shown to have an influence on the membrane lipid composition of microorganisms (Männistö and Puhakka, 2001; Elling et al., 2014). Thus, in order to reliably compare the influence of temperature and pH on the distribution of 3-OH FAs between the different strains, the bacterial biomass was collected at the same stage of growth. For all the strains, the biomass was collected during the exponential phase by dividing by two the OD obtained at the stationary phase (reference OD). After subculturing as described above, the final cultures were performed in triplicate in a final volume of 150 mL of sterile R2A medium incubated at 180 rpm until reaching the reference OD. The cells were collected in 50 mL Falcon^®^ tubes and centrifuged at 7,000 rpm (JA12 rotor, Beckman) for 10 min at 20°C. Each bacterial pellet was resuspended in 10 mL of 0.9 g/L NaCl to remove traces of R2A medium and then centrifuged again at the same speed as before. The supernatant was removed and the cell pellets were frozen at −20°C before being freeze-dried for 48 h at −80°C at a pressure of 0.93 Pa. The freeze-dried cell pellets were kept frozen at −20°C before lipid extraction and analysis.

### 2.3. Lipid extraction

The freeze-dried cells were first subjected to acid methanolysis in 1 M HCl /MeOH at 100°C for 3 h. The suspension was then centrifuged at 15°C and 3,000 rpm for 5 min. The supernatant was collected in an Erlenmeyer flask. The pellet was extracted 3 times with a mixture of dichloromethane (DCM):MeOH (1:1, v/v; 25 mL). Each extraction was followed by centrifugation and pooling of all extracts. The extracts were then transferred to a separation funnel and washed with ultrapure water in order to neutralize the organic phase. The organic phase was recovered in a new Erlenmeyer flask. The aqueous phase was extracted twice with DCM (20 mL). The organic phase was recovered and pooled with the organic phase previously obtained. The organic phase containing the lipids was dried with sodium sulfate, then rotary-evaporated, diluted with a mixture of DCM:MeOH (5:1), transferred into a 4 mL vial and dried under nitrogen flow. The lipid extract was resuspended in 1 mL DCM and stored at −20°C until 3-OH FA analysis.

### 2.4. Lipid analysis

The lipid extracts were first derivatised with a solution of *N,O*- bis(trimethylsilyl)trifluoroacetamide (BSTFA) at 70°C for 45 min. Fatty acids were analyzed by gas chromatography coupled to mass spectrometry using an Agilent Network 6980 GC System coupled with a 5973 Mass Selective Detector, with electron impact at 70 eV. A Restek RXI-5 Sil MS silica column (60 m × 0.25 mm, i.d. 0.50 μm film thickness) was used with He as the carrier gas at 1 mL/min, as previously described (Huguet et al., 2019). The GC oven program was: 70°C to 200°C at 10°C/min, then to 310°C (held 20 min) at 2°C/min. Samples were injected in splitless mode and the injector was set at 280°C. The individual fatty acids (either hydroxy or non-hydroxy homologues) were first identified from the total ion chromatogram (TIC). The corresponding peaks were integrated, allowing the determination of the relative abundances of the different fatty acids.

A focus was then placed on the different 3-OH FAs in the characteristic *m/z* 175 mass trace chromatogram (*cf.* Huguet et al., 2019), allowing the identification of the low-abundant homologues (not visible in the TIC) based on their relative retention times and mass spectra. The individual 3-OH FAs were semi-quantified by integrating the respective peak on the *m/z* 175 ion chromatogram and comparing the area with an analogous deuterated injection standard (3-hydroxytetradecanoic acid, 2,2,3,4,4-d5; Sigma-Aldrich, France), as previously described (Huguet et al., 2019). The injection standard (0.5 mg/mL) was added just before analysis, with a proportion of 3 μL of standard to 100 μL of sample, as detailed by Huguet et al. (2019). The *m/z* 178 fragment was used for identification and integration of the deuterated internal standard.

The RIAN index was calculated as follows in the range C_10_-C_18_ (Wang et al., 2016):


(1) RIAN=−log[(I+A)/N]

where I, A, N represent the sum of all *iso*, *anteiso* and *normal* 3-OH FAs, respectively.

RAN_15_ and RAN_17_ indices were calculated as follows (Wang et al., 2016):


(2) $${\mathrm{RAN}}_{15}=\left[anteiso-C_{15}\right]/\left[normal-C_{15}\right]$$

(3) $${\mathrm{RAN}}_{17}=\left[anteiso-C_{17}\right]/\left[normal-C_{17}\right]$$

### 2.5. Statistical analyses

All the statistical analyses were performed using R (v3.6.3, R Core Team, 2020). To investigate the statistical differences in the relative abundances of 3-OH FAs depending on the pH or temperature of the culture experiments, analysis of variance (ANOVA) and post-hoc Tukey tests were used. In order to investigate the correlations between the temperature or pH and the relative abundances of 3-OH FAs, pairwise Spearman correlation matrices and single linear regressions were performed for each strain. The *value of p* was set at 0.05 for all the statistical analyses.

## 3. Results

### 3.1. Global distribution of fatty acids in bacterial strains isolated from soils

#### 3.1.1. Non-hydroxy fatty acids

The membrane lipid profiles of the three strains cultivated under different temperature and pH condition were evaluated (Tables 1–3). The non-hydroxy FAs were generally more abundant than 3-OH FAs, especially in strain A, where non-hydroxy FAs represented more than 70% of the total relative abundance in FAs underdifferent cultivation conditions (FAs; Table 1). For strains B and C, non-hydroxy FAs were generally slightly more abundant (> 55% of total FAs) than 3-OH FAs. Straight-chain (i.e., *normal*) monounsaturated FAs were the major group of non-hydroxy FAs in strains B and C (Tables 2, 3). In contrast, in strain A, the branched saturated FAs were as abundant as the *normal* monounsaturated FAs (Table 1). Strain A also differed from strains B and C by the presence of branched unsaturated FAs (*i*-C_15:1_ and to a lesser extent *i*-C_16:1_; Table 1). At the individual level, *n*-C_16:1_ was observed to be the dominant non-hydroxy FA in strains B and C, followed, to a lesser extent, by *n*-C_16:0_, *n*-C_18:0_ and *i*-C_15:0_ (Tables 2, 3). Regarding strain A, the most abundant non-hydroxy FAs were the *i*-C_15:0_ and *n*-C_16:1_ homologues, followed by the *n*-C_16:0_ and a couple of FAs in the same range such as *n*-C_15:0,_-C_17:1_ and *i*-C_15:1_.

**Table 1:** Relative percentage abundances of non-hydroxy fatty acids vs. total fatty acids for strain A under different temperature conditions (at pH 8) and at different pH conditions (at 25°C).

Strain A	Temperature conditions (°C)							pH conditions					
	5°C	10°C	15°C	20°C	25°C	*R* ^2^	Value of *p*	5	6	7	8	*R* ^2^	Value of *p*
Normal saturated													
15:0	3.08 ± 0.16	4.60 ± 0.18	5.59 ± 0.42	7.45 ± 0.57	9.15 ± 1.10	**0.94**	2.11E-09	8.41 ± 3.27	12.39 ± 0.53	10.34 ± 2.34	9.15 ± 1.10	8.14E-05	0.98
16:0	8.75 ± 5.91	6.25 ± 1.29	6.13 ± 0.92	8.43 ± 0.74	12.81 ± 2.84	0.17	0.12	7.93 ± 2.36	7.50 ± 0.41	7.76 ± 0.70	12.81 ± 2.84	**0.38**	0.032
18:0	2.39 ± 0.83	3.71 ± 0.46	1.57 ± 0.76	1.27 ± 0.49	2.84 ± 0.42	0.05	0.45	0.86 ± 0.16	0.75 ± 0.28	0.62 ± 0.07	2.84 ± 0.42	**0.48**	0.013
Total	14.21 ± 5.83	14.57 ± 1.78	13.30 ± 1.50	17.17 ± 0.77	24.80 ± 4.11	**0.45**	6.29E-03	17.21 ± 5.44	20.62 ± 0.33	18.73 ± 2.95	24.80 ± 4.11	0.31	0.06
Branched saturated													
iso 14:0	0.63 ± 0.33	0.47 ± 0.03	0.19 ± 0.32	n.d.	0.68 ± 0.02	0.05	0.42	0.77 ± 0.26	0.42 ± 0.03	0.47 ± 0.05	0.68 ± 0.02	0.02	0.65
iso 15:0	18.34 ± 0.37	19.27 ± 0.81	22.86 ± 3.19	24.18 ± 0.55	22.77 ± 1.65	**0.54**	1.75E-03	26.47 ± 0.93	25.37 ± 1.24	30.48 ± 1.16	22.77 ± 1.65	0.05	0.48
anteiso 15:0	5.89 ± 0.07	4.59 ± 0.25	4.01 ± 0.43	2.67 ± 0.14	2.20 ± 0.20	**0.96**	3.88E-10	1.07 ± 0.11	2.06 ± 1.72	1.54 ± 0.15	2.20 ± 0.20	0.15	0.22
iso 16:0	1.07 ± 0.31	1.48 ± 0.19	1.19 ± 0.21	0.86 ± 0.75	0.98 ± 0.03	0.06	0.37	0.09 ± 0.16	n.d.	n.d.	0.98 ± 0.03	n.d.	n.d.
Total	25.94 ± 0.73	25.82 ± 1.22	28.24 ± 3.11	27.71 ± 1.08	26.63 ± 1.85	0.07	0.34	28.40 ± 1.19	27.85 ± 1.10	32.50 ± 1.27	26.63 ± 1.85	8.33E-04	0.93
Normal unsaturated													
15:1	1.41 ± 0.19	1.93 ± 0.18	1.92 ± 0.11	1.90 ± 0.09	1.86 ± 0.25	0.25	0.057	3.77 ± 0.70	2.70 ± 2.09	2.31 ± 0.41	1.86 ± 0.25	**0.35**	0.045
16:1	15.61 ± 0.35	15.58 ± 0.70	17.85 ± 1.34	17.88 ± 1.13	18.08 ± 0.63	**0.58**	1.05E-03	16.02 ± 2.56	15.49 ± 0.80	18.85 ± 0.87	18.08 ± 0.63	**0.34**	0.047
17:1	7.47 ± 2.93	10.86 ± 0.91	8.93 ± 0.81	4.95 ± 2.85	3.87 ± 1.81	**0.37**	0.017	4.00 ± 2.20	4.27 ± 0.87	3.15 ± 0.64	3.87 ± 1.81	0.017	0.69
18:1	n.d.	n.d.	n.d.	n.d.	n.d.	n.d.	n.d.	n.d.	n.d.	n.d.	n.d.	n.d.	n.d.
Total	24.49 ± 3.29	28.37 ± 0.75	28.45 ± 2.23	24.74 ± 2.45	23.81 ± 1.10	0.07	0.34	23.80 ± 1.59	22.46 ± 2.10	24.32 ± 1.89	23.81 ± 1.10	0.019	0.67
Branched unsaturated													
iso 15:1	9.73 ± 0.43	8.16 ± 0.44	6.96 ± 0.67	4.54 ± 0.19	4.04 ± 0.14	**0.95**	8.90E-10	3.41 ± 0.38	3.23 ± 0.14	3.39 ± 0.23	4.04 ± 0.14	**0.38**	0.032
iso 16:1	0.22 ± 0.38	0.48 ± 0.42	0.24 ± 0.41	n.d.	n.d.	**0.56**	1.27E-03	n.d.	n.d.	n.d.	n.d.	0.09	0.14
Total	9.95 ± 0.54	8.64 ± 0.36	7.20 ± 0.33	4.54 ± 0.19	4.04 ± 0.14	**0.95**	4.29E-10	3.41 ± 0.38	3.23 ± 0.14	3.39 ± 0.23	4.04 ± 0.14	**0.38**	0.03
Total non-hydroxy FAs	74.60 ± 2.86	77.40 ± 2.90	77.18 ± 5.06	74.15 ± 3.22	79.28 ± 5.00	0.05	0.41	72.81 ± 5.95	74.16 ± 1.55	78.93 ± 5.83	79.28 ± 5.00	0.30	0.065

Data are means of three biological replicates. Values after (±) are standard deviations. Correlations of the relative abundances of each fatty acid (FA) with either temperature or pH are provided with corresponding *p*-values. Significant correlations (*p* < 0.005) are indicated in bold. n.d.: not detected. Strain A: *Flavobacterium pectinovorum* (98.78% similarity).

**Table 2:** Relative percentage abundances of non-hydroxy fatty acids vs. total fatty acids for strain B under different temperature conditions (at pH 5) and at different pH conditions (at 25°C).

Strain B	Temperature conditions (°C)							pH conditions					
	5°C	10°C	15°C	20°C	25°C	*R* ^2^	Value of *p*	5	6	7	8	*R* ^2^	Value of *p*
Normal saturated													
15:0	n.d.	n.d.	n.d.	n.d.	n.d.	n.d.	n.d.	n.d.	n.d.	n.d.	n.d.	n.d.	n.d.
16:0	11.80 ± 0.99	13.18 ± 3.99	12.13 ± 0.98	11.31 ± 1.59	12.09 ± 1.44	0.010	0.73	12.09 ± 1.44	13.94 ± 4.77	12.69 ± 1.02	17.08 ± 1.84	**0.27**	0.081
18:0	4.94 ± 1.54	5.90 ± 3.17	3.42 ± 0.41	6.19 ± 3.21	2.39 ± 0.62	**0.23**	0.28	2.39 ± 0.62	1.87 ± 0.87	2.40 ± 0.64	3.62 ± 0.67	0.30	0.067
Total	16.74 ± 2.53	19.07 ± 5.50	15.56 ± 1.39	17.50 ± 4.80	14.48 ± 2.06	0.83	0.35	14.48 ± 2.06	15.82 ± 3.91	15.08 ± 1.35	20.70 ± 2.28	0.38	0.032
Branched saturated													
iso 14:0	n.d.	2.80 ± 2.52	n.d.	n.d.	0.13 ± 0.22	n.d.	n.d.	0.13 ± 0.22	0.31 ± 0.53	0.22 ± 0.38	n.d.	0.26	0.092
iso 15:0	5.61 ± 0.37	7.09 ± 1.08	6.03 ± 0.32	7.27 ± 0.61	10.10 ± 1.00	**0.59**	7.83E-04	10.10 ± 1.00	6.35 ± 0.99	6.50 ± 1.22	9.23 ± 2.24	0.02	0.68
anteiso 15:0	2.52 ± 0.22	1.72 ± 1.54	1.49 ± 0.16	0.25 ± 0.44	0.23 ± 0.40	**0.81**	4.84E-06	0.23 ± 0.40	n.d.	0.14 ± 0.24	n.d.	**n.d.**	1.30E-02
iso 16:0	n.d.	n.d.	n.d.	n.d.	n.d.	n.d.	n.d.	n.d.	n.d.	n.d.	n.d.	n.d.	n.d.
Total	8.13 ± 0.59	11.60 ± 1.37	7.52 ± 0.20	7.53 ± 0.73	10.45 ± 1.62	0.002	0.88	10.45 ± 1.62	6.65 ± 0.48	6.85 ± 0.91	9.23 ± 2.24	0.04	0.55
Normal unsaturated													
15:1	n.d.	n.d.	n.d.	n.d.	n.d.	n.d.	n.d.	n.d.	n.d.	n.d.	n.d.	n.d.	n.d.
16:1	33.69 ± 1.43	33.71 ± 1.87	32.65 ± 2.33	33.11 ± 2.59	30.86 ± 1.29	0.21	0.083	30.86 ± 1.29	33.05 ± 1.30	31.54 ± 2.03	7.70 ± 0.78	**0.57**	4.68E-03
17:1	7.89 ± 1.70	4.49 ± 1.41	2.33 ± 0.28	n.d.	0.57 ± 0.52	**0.77**	1.85E-05	0.57 ± 0.52	0.46 ± 0.09	0.71 ± 0.05	n.d.	**0.05**	0.49
18:1	0.36 ± 0.34	n.d.	n.d.	n.d.	0.32 ± 0.29	0.09	0.29	0.32 ± 0.29	3.18 ± 5.50	n.d.	n.d.	n.d.	n.d.
Total	41.94 ± 0.38	38.20 ± 0.78	34.99 ± 2.04	33.11 ± 2.59	31.76 ± 2.05	**0.83**	2.31E-06	31.76 ± 2.05	36.69 ± 6.62	32.25 ± 1.99	7.70 ± 0.78	0.53	7.10E-03
Branched unsaturated													
iso 15:1	n.d.	n.d.	n.d.	n.d.	n.d.	n.d.	n.d.	n.d.	n.d.	n.d.	n.d.	n.d.	n.d.
Iso 16:1	n.d.	n.d.	n.d.	n.d.	n.d.	n.d.	n.d.	n.d.	n.d.	n.d.	n.d.	n.d.	n.d.
Total	n.d.	n.d.	n.d.	n.d.	n.d.	n.d.	n.d.	n.d.	n.d.	n.d.	n.d.	n.d.	n.d.
Total non-hydroxy FAs	66.82 ± 2.89	68.89 ± 6.23	58.06 ± 3.62	58.13 ± 6.64	56.69 ± 5.08	**0.45**	6.54E-03	56.69 ± 5.08	59.16 ± 10.09	54.18 ± 3.91	37.62 ± 0.10	**0.51**	9.20E-03

Data are means of three biological replicates. Values after (±) are standard deviations. Correlations of the relative abundances of each fatty acid (FA) with either temperature or pH are provided with corresponding *p*-values. Significant correlations (*p* < 0.005) are indicated in bold. n.d.: not detected. Strain B: *Pedobacter lusitanus* NL19 (98.54% similarity).

**Table 3:** Relative percentage abundances of non-hydroxy fatty acids vs. total fatty acids for strain C under different temperature conditions (at pH 5) and at different pH conditions (at 25°C).

Strain C	Temperature conditions (°C)							pH conditions					
	5°C	10°C	15°C	20°C	25°C	*R* ^2^	Value of *p*	5	6	7	8	*R* ^2^	Value of *p*
Normal saturated													
15:0	n.d.	0.17 ± 0.29	n.d.	n.d.	n.d.	0.07	0.23	n.d.	0.35 ± 0.30	0.37 ± 0.32	0.48 ± 0.42	**0.41**	1.94E-05
16:0	11.82 ± 0.78	9.02 ± 1.96	7.06 ± 1.29	8.17 ± 0.72	6.69 ± 1.12	**0.56**	1.32E-03	6.69 ± 1.12	8.03 ± 1.97	18.15 ± 5.54	9.39 ± 2.66	0.15	0.21
18:0	10.58 ± 1.06	4.08 ± 0.81	1.85 ± 0.39	5.76 ± 0.59	6.20 ± 0.68	0.12	0.21	6.20 ± 0.68	1.28 ± 0.07	2.74 ± 1.13	1.47 ± 1.29	**0.45**	0.016
Total	22.40 ± 1.58	13.27 ± 2.55	8.91 ± 1.67	13.93 ± 1.14	12.89 ± 1.68	**0.31**	0.031	12.89 ± 1.68	9.31 ± 1.94	21.26 ± 5.38	11.34 ± 3.69	0.02	0.64
Branched saturated													
iso 14:0	n.d.	0.57 ± 0.52	0.33 ± 0.29	n.d.	0.66 ± 0.11	0.07	0.33	0.66 ± 0.11	0.35 ± 0.30	n.d.	n.d.	n.d.	n.d.
iso 15:0	5.13 ± 0.24	4.28 ± 3.71	5.29 ± 0.93	7.06 ± 3.48	8.96 ± 3.64	0.26	0.053	8.96 ± 3.64	8.00 ± 2.11	8.99 ± 0.54	11.69 ± 1.07	0.21	0.14
anteiso 15:0	2.20 ± 0.23	3.85 ± 3.45	0.82 ± 0.73	n.d.	0.45 ± 0.02	0.23	0.070	0.45 ± 0.02	n.d.	0.53 ± 0.04	0.63 ± 0.04	**0.85**	2.18E-05
iso 16:0	n.d.	n.d.	n.d.	n.d.	n.d.	n.d.	n.d.	n.d.	n.d.	n.d.	n.d.	n.d.	n.d.
Total	7.33 ± 0.29	8.70 ± 0.76	6.44 ± 1.02	7.06 ± 3.48	10.06 ± 3.56	0.06	0.40	10.06 ± 3.56	8.35 ± 2.23	9.34 ± 0.85	12.33 ± 1.12	0.14	0.23
Normal unsaturated													
15:1	n.d.	n.d.	n.d.	n.d.	n.d.	0.03	0.41	n.d.	n.d.	n.d.	0.22 ± 0.38	n.d.	n.d.
16:1	26.58 ± 1.29	30.16 ± 4.05	26.96 ± 2.18	33.36 ± 5.15	29.27 ± 4.60	0.10	0.261	29.27 ± 4.60	32.75 ± 3.00	32.22 ± 1.98	38.65 ± 4.60	**0.46**	0.016
17:1	3.26 ± 1.52	5.64 ± 0.66	2.77 ± 2.42	0.95 ± 1.63	2.04 ± 1.12	0.24	0.062	2.04 ± 1.12	2.39 ± 0.13	2.95 ± 0.32	4.19 ± 0.47	**0.66**	1.32E-03
18:1	n.d.	n.d.	n.d.	1.02 ± 1.77	n.d.	0.07	0.21	n.d.	n.d.	0.33 ± 0.05	0.64 ± 0.16	n.d.	n.d.
Total	29.84 ± 0.29	35.80 ± 3.97	29.73 ± 0.68	35.33 ± 7.20	31.31 ± 3.52	0.007	0.77	31.31 ± 3.52	35.14 ± 2.90	35.40 ± 2.41	43.49 ± 5.12	**0.59**	3.33E-03
Branched unsaturated													
iso 15:1	n.d.	n.d.	n.d.	n.d.	n.d.	n.d.	n.d.	n.d.	n.d.	n.d.	n.d.	n.d.	n.d.
iso 16:1	n.d.	0.21 ± 0.37	n.d.	n.d.	n.d.	0.07	0.25	n.d.	n.d.	n.d.	n.d.	n.d.	n.d.
Total	n.d.	0.21 ± 0.37	n.d.	n.d.	n.d.	n.d.	n.d.	n.d.	n.d.	n.d.	n.d.	n.d.	n.d.
Total non-hydroxy FAs	59.58 ± 1.46	57.99 ± 5.08	45.08 ± 0.48	56.32 ± 7.35	54.27 ± 8.56	0.06	0.36	54.27 ± 8.56	52.80 ± 5.43	66.01 ± 3.85	67.16 ± 3.07	**0.52**	7.74E-03

Data are means of three biological replicates. Values after (±) are standard deviations. Correlations of the relative abundances of each fatty acid (FA) with either temperature or pH are provided with corresponding *p*-values. Significant correlations (*p* < 0.005) are indicated in bold. n.d.: not detected. Strain C: *Pedobacter panaciterrae* (99.27% similarity).

#### 3.1.2. 3-OH FAs

**Figure 1:**
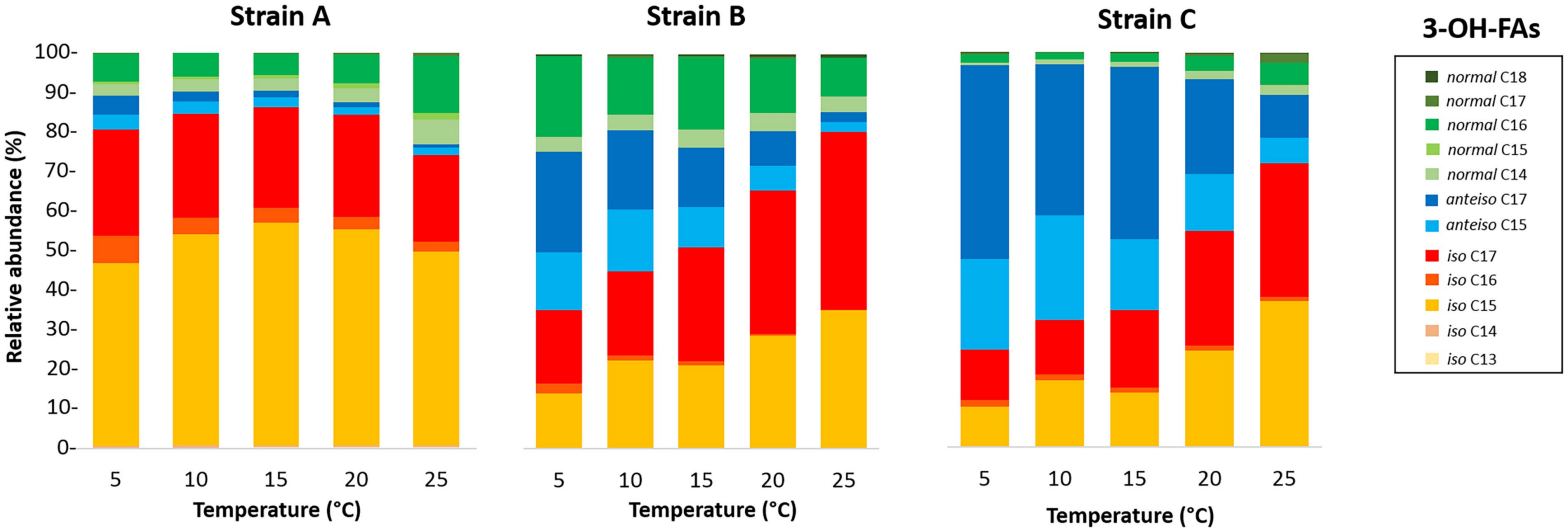
Variation of the relative abundances of 3-OH-FAs with temperature for the three *Bacteroidetes* strains isolated from different altitudinal soils of the French Alps. Strain A: *Flavobacterium pectinovorum* (98.78% similarity). Strain B: *Pedobacter lusitanus* NL19 (98.54% similarity). Strain C: *Pedobacter panaciterrae* (99.27% similarity).

**Figure 2:**
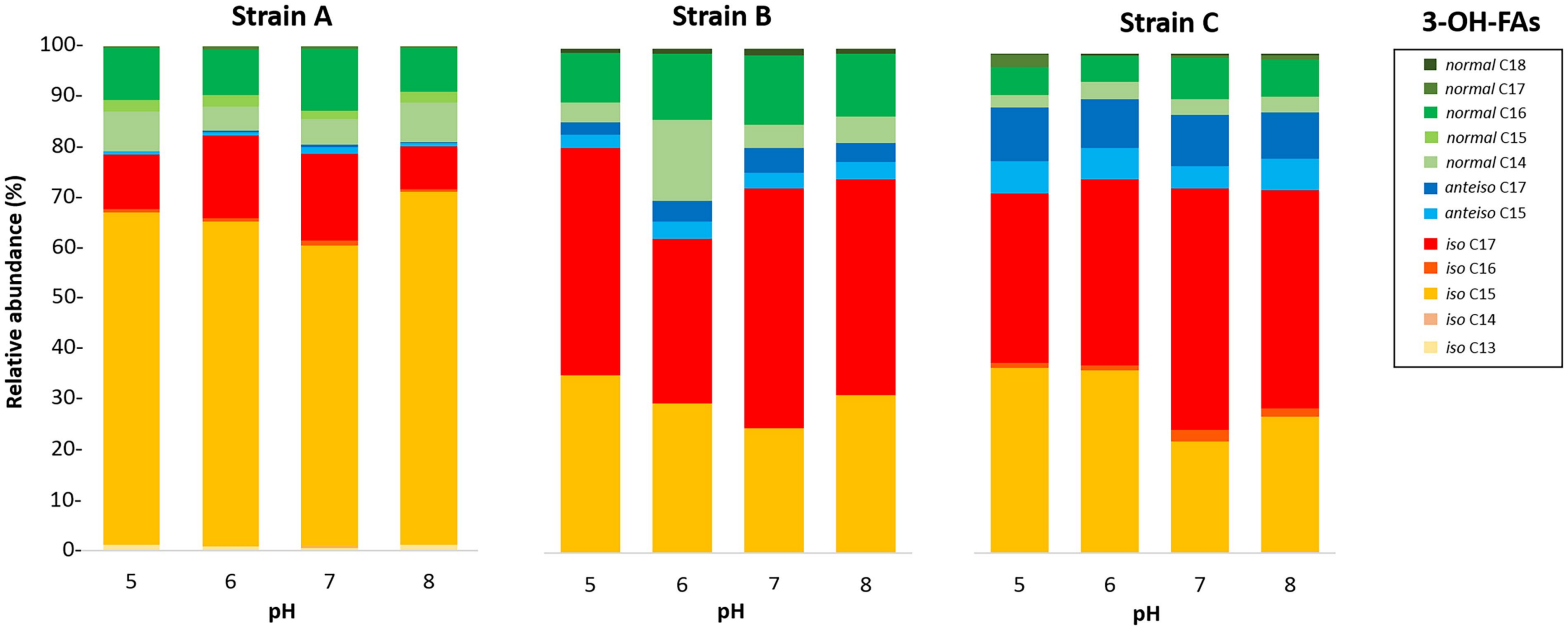
Variation of the relative abundances of 3-OH FAs with pH for the three *Bacteroidetes* strains isolated from different altitudinal soils of the French Alps. Strain A: *Flavobacterium pectinovorum* (98.78% similarity). Strain B: *Pedobacter lusitanus* NL19 (98.54% similarity). Strain C: *Pedobacter panaciterrae* (99.27% similarity).

The distribution of the 3-OH FAs was also examined in detail (Figure 1; Tables 4–6). The detected 3-OH FAs showed a carbon number range between 13 and 18 carbon atoms. Independently from the different conditions in the temperature and pH cultivation experiments (Figures 1, 2), the *iso* homologues were the most abundant 3-OH FAs in strain A (Table 4). This was also the case for strain B, except at a growth temperature of 5°C (Table 5), where the *anteiso* homologues were slightly more abundant than the *iso* homologues. In addition, the relative abundance of the *normal* homologues was higher than the one of the *anteiso* 3-OH FAs for strain A (Table 4), whereas an opposite trend was observed for strain C (Table 6). In strain C the *iso* homologues were also the most abundant whatever the pH (Figure 2), but in the temperature experiments they only prevailed in the cultivations at or higher than 20°C, while in cultivations at lower temperature *anteiso* 3-OH FAs dominated (Figure 1). Regarding strain B, the relative abundance of *normal* vs. *anteiso* 3-OH FAs was highly dependent on the cultivation conditions (Table 4).

**Table 4:** Relative percentage abundances of 3-hydroxy fatty acids vs. total 3-hydroxy fatty acids and related indices [defined by Wang et al. (2016)] for strain A cultivated under different temperature (at pH 8) and pH (at 25°C) conditions.

Strain A	Temperature conditions (°C)							pH conditions					
	5°C	10°C	15°C	20°C	25°C	*R* ^2^	Value of *p*	5	6	7	8	*R* ^2^	Value of *p*
iso C_13_	0.15 ± 0.006	0.28 ± 0.02	0.33 ± 0.06	0.42 ± 0.03	0.48 ± 0.03	**0.91**	2.59E-08	1.15 ± 0.36	0.79 ± 0.08	0.54 ± 0.21	0.48 ± 0.03	**0.64**	1.73E-03
iso C_14_	0.52 ± 0.03	0.45 ± 0.04	0.44 ± 0.15	0.37 ± 0.04	0.31 ± 0.001	**0.55**	1.45E-03	n.d.	0.09 ± 0.08	0.21 ± 0.07	0.31 ± 0.001	**0.88**	7.93E-06
normal C_14_	2.88 ± 0.15	3.06 ± 0.45	3.22 ± 0.31	3.69 ± 0.08	6.35 ± 0.88	**0.64**	3.51E-04	7.81 ± 2.25	4.66 ± 0.52	5.03 ± 1.05	6.35 ± 0.88	0.07	0.39
iso C_15_	46.17 ± 1.43	53.41 ± 5.32	56.37 ± 10.02	54.64 ± 4.40	49.02 ± 6.17	0.02	0.58	65.92 ± 7.93	64.32 ± 4.78	59.69 ± 9.43	49.02 ± 6.17	**0.48**	0.012
anteiso C_15_	3.74 ± 0.16	3.08 ± 0.26	2.45 ± 0.29	1.88 ± 0.22	1.98 ± 0.19	**0.85**	1.10E-6	0.61 ± 0.11	0.71 ± 0.09	1.28 ± 0.34	1.98 ± 0.19	**0.84**	2.74E-05
normal C_15_	0.72 ± 0.008	0.79 ± 0.08	0.81 ± 0.28	1.10 ± 0.05	1.60 ± 0.08	**0.73**	4.78E-05	2.35 ± 0.32	2.41 ± 0.03	1.61 ± 0.28	1.60 ± 0.08	**0.64**	1.79E-03
iso C_16_	6.92 ± 0.62	4.27 ± 0.89	3.77 ± 0.28	3.14 ± 0.54	2.47 ± 0.68	**0.68**	1.42E-04	0.58 ± 0.35	0.71 ± 0.10	1.07 ± 0.32	2.47 ± 0.68	**0.67**	1.09E-03
normal C_16_	7.10 ± 0.21	5.88 ± 0.76	5.53 ± 2.43	7.44 ± 1.64	14.45 ± 2.69	**0.41**	9.94E-03	10.20 ± 1.90	8.94 ± 1.58	12.29 ± 5.48	14.45 ± 2.69	0.28	0.078
iso C_17_	26.93 ± 0.79	26.30 ± 3.79	25.40 ± 3.92	25.95 ± 2.23	21.89 ± 3.66	0.23	0.073	10.84 ± 7.68	16.33 ± 3.67	17.17 ± 5.92	21.89 ± 3.66	**0.41**	0.026
anteiso C_17_	4.81 ± 0.30	2.44 ± 0.53	1.62 ± 0.86	1.05 ± 0.19	0.75 ± 0.23	**0.79**	8.29E-06	0.13 ± 0.12	0.32 ± 0.05	0.56 ± 0.13	0.75 ± 0.23	**0.79**	1.07E-04
normal C_17_	0.07 ± 0.06	0.04 ± 0.07	0.07 ± 0.12	0.32 ± 0.04	0.70 ± 0.05	**0.71**	7.94E-05	0.42 ± 0.37	0.72 ± 0.13	0.54 ± 0.07	0.70 ± 0.05	0.14	0.23
normal C_18_	n.d.	n.d.	n.d.	n.d.	n.d.	n.d.	n.d.	n.d.	n.d.	n.d.	n.d.	n.d.	n.d.
Total iso 3-OH FAs	80.69 ± 0.16	84.71 ± 0.74	86.31 ± 4.27	84.52 ± 1.75	74.17 ± 1.88	0.16	0.15	78.49 ± 1.03	82.24 ± 1.18	78.69 ± 3.85	74.17 ± 1.88	0.29	0.070
Total anteiso 3-OH FAs	8.55 ± 0.13	5.52 ± 0.29	4.07 ± 1.15	2.93 ± 0.35	2.72 ± 0.05	**0.85**	1.15E-06	0.73 ± 0.02	1.04 ± 0.05	1.84 ± 0.35	2.72 ± 0.05	**0.93**	5.20E-07
Total normal 3-OH FAs	10.77 ± 0.13	9.77 ± 0.45	9.62 ± 3.13	12.56 ± 1.61	23.10 ± 1.86	**0.54**	1.81E-03	20.78 ± 1.04	16.72 ± 1.22	19.47 ± 4.20	23.10 ± 1.86	0.12	0.25
Iso/anteiso 3-OH FAs	9.44 ± 0.15	15.39 ± 0.97	22.42 ± 6.39	29.16 ± 3.75	27.27 ± 1.06	**0.79**	1.05E-05	107.0 ± 2.81	79.48 ± 3.30	43.57 ± 7.11	27.27 ± 1.06	**0.97**	6.22E-09
Iso/normal 3-OH FAs	7.50 ± 0.10	8.68 ± 0.49	9.65 ± 3.17	6.82 ± 1.06	3.23 ± 0.36	**0.34**	0.023	3.79 ± 0.23	4.94 ± 0.45	4.19 ± 1.06	3.23 ± 0.36	0.11	0.28
Anteiso/normal 3-OH FAs	0.79 ± 0.02	0.56 ± 0.005	0.43 ± 0.03	0.24 ± 0.04	0.12 ± 0.009	**0.98**	6.79E-13	0.04 ± 0.02	0.06 ± 0.008	0.10 ± 0.04	0.12 ± 0.009	**0.79**	1.05E-04
RIAN	−0.92 ± 0.006	−0.97 ± 0.02	−0.99 ± 0.15	−0.85 ± 0.07	−0.52 ± 0.05	**0.51**	2.87E-03	−0.58 ± 0.03	−0.70 ± 0.04	−0.62 ± 0.12	−0.52 ± 0.05	0.11	0.29
RAN_15_	5.22 ± 0.27	3.96 ± 0.75	3.17 ± 0.67	1.70 ± 0.16	1.24 ± 0.14	**0.92**	2.31E-08	0.26 ± 0.07	0.30 ± 0.04	0.79 ± 0.08	1.24 ± 0.14	**0.88**	5.57E-06
RAN_17_	44.80 ± 2.33	24.44	12.51	3.30 ± 0.21	1.07 ± 0.34	**0.92**	1.26E-08	0.31 ± 0.03	0.46 ± 0.02	1.02 ± 0.10	1.07 ± 0.34	**0.73**	4.20E-04

Data are means of three biological replicates. Values after (±) are standard deviations. Correlations of the relative abundances of each 3-hydroxy fatty acid with either temperature or pH are provided with corresponding *p*-values. Significant correlations (*p* < 0.005) are indicated in bold. n.d.: not detected. Strain A: *Flavobacterium pectinovorum* (98.78% similarity).

**Table 5:** Relative percentage abundances of 3-hydroxy fatty acids vs. total 3-hydroxy fatty acids and related indices [defined by Wang et al. (2016)] for strain B cultivated under different temperature (at pH 5) and pH (at 25°C) conditions.

Strain B	Temperature conditions (°C)							pH conditions					
	5°C	10°C	15°C	20°C	25°C	*R* ^2^	Value of *p*	5	6	7	8	*R* ^2^	Value of *p*
iso C_13_	n.d.	n.d.	n.d.	n.d.	n.d.	n.d.	n.d.	n.d.	n.d.	n.d.	n.d.	n.d.	n.d.
iso C_14_	0.19 ± 0.06	n.d.	n.d.	n.d.	n.d.	n.d.	n.d.	n.d.	n.d.	n.d.	n.d.	n.d.	n.d.
normal C_14_	3.70 ± 0.62	3.89 ± 0.47	4.57 ± 1.04	4.66 ± 1.12	4.08 ± 1.18	0.07	0.36	4.08 ± 1.18	16.07 ± 17.17	4.53 ± 0.51	5.37 ± 0.46	0.010	0.76
iso C_15_	13.93 ± 3.19	22.37 ± 0.47	21.24 ± 3.39	28.68 ± 6.91	35.18 ± 5.23	**0.72**	6.29E-05	35.18 ± 5.23	29.61 ± 0.85	24.80 ± 3.99	31.27 ± 4.10	0.08	0.35
anteiso C_15_	14.61 ± 3.09	15.57 ± 1.77	10.24 ± 2.26	6.31 ± 1.73	2.55 ± 0.64	**0.82**	3.12E-06	2.55 ± 0.64	3.56 ± 0.85	3.15 ± 0.80	3.43 ± 0.25	0.14	0.24
normal C_15_	n.d.	n.d.	n.d.	n.d.	n.d.	n.d.	n.d.	n.d.	n.d.	n.d.	n.d.	n.d.	n.d.
iso C_16_	2.38 ± 0.27	1.40 ± 0.30	0.88 ± 0.11	0.37 ± 0.14	0.03 ± 0.05	**0.93**	9.88E-09	0.03 ± 0.05	n.d.	n.d.	n.d.	n.d.	n.d.
normal C_16_	20.44 ± 1.59	14.45 ± 1.26	18.51 ± 1.48	13.87 ± 2.03	9.77 ± 0.30	**0.62**	4.59E-04	9.77 ± 0.30	13.11 ± 1.98	13.71 ± 0.91	12.34 ± 0.95	0.27	0.08
iso C_17_	18.76 ± 1.50	21.28 ± 2.06	29.04 ± 2.73	36.42 ± 6.20	45.15 ± 6.57	**0.86**	7.20E-07	45.15 ± 6.57	32.63 ± 4.49	47.44 ± 4.38	42.78 ± 2.62	0.016	0.70
anteiso C_17_	25.53 ± 3.49	20.25 ± 1.95	14.96 ± 2.35	8.71 ± 1.39	2.43 ± 0.44	**0.95**	5.19E-10	2.43 ± 0.44	4.02 ± 0.46	4.99 ± 0.49	3.79 ± 0.50	0.32	0.053
normal C_17_	0.18 ± 0.08	0.54 ± 0.94	0.16 ± 0.06	0.36 ± 0.14	0.07 ± 0.06	0.02	0.61	0.07 ± 0.06	n.d.	n.d.	n.d.	**0.36**	0.039
normal C_18_	0.29 ± 0.02	0.25 ± 0.21	0.40 ± 0.005	0.63 ± 0.09	0.74 ± 0.15	**0.70**	1.08E-03	0.74 ± 0.15	1.01 ± 0.29	1.37 ± 0.13	1.02 ± 0.12	0.25	0.097
Total iso 3-OH FAs	35.26 ± 1.50	45.05 ± 1.65	51.16 ± 0.60	65.47 ± 0.71	80.36 ± 1.31	**0.97**	2.19E-11	80.36 ± 1.31	62.23 ± 13.74	72.25 ± 1.61	74.05 ± 1.70	0.01	0.72
Total anteiso 3-OH FAs	40.13 ± 0.41	35.82 ± 1.04	25.20 ± 0.09	15.02 ± 0.40	4.97 ± 0.52	**0.98**	7.88E-13	4.97 ± 0.52	7.58 ± 1.24	8.14 ± 0.76	7.22 ± 0.27	**0.36**	0.04
Total normal 3-OH FAs	24.60 ± 1.11	19.13 ± 1.35	23.64 ± 0.52	19.51 ± 0.95	14.66 ± 0.95	**0.57**	1.12E-03	14.66 ± 0.95	30.18 ± 14.97	19.61 ± 1.07	18.73 ± 1.43	4.7E-04	0.95
Iso/Anteiso 3-OH FAs	0.88 ± 0.05	1.26 ± 0.07	2.03 ± 0.03	4.36 ± 0.11	16.29 ± 1.97	**0.68**	1.69E-04	16.29 ± 1.97	8.15 ± 0.55	8.94 ± 0.06	10.28 ± 0.62	**0.33**	0.05
Iso/normal 3-OH FAs	1.44 ± 0.13	2.37 ± 0.25	2.17 ± 0.07	3.36 ± 0.20	5.50 ± 0.44	**0.82**	3.23E-06	5.50 ± 0.44	2.50 ± 1.32	3.69 ± 0.27	3.97 ± 0.39	0.10	0.33
Anteiso/normal 3-OH FAs	1.63 ± 0.06	1.88 ± 0.15	1.07 ± 0.02	0.77 ± 0.06	0.34 ± 0.03	**0.86**	7.36E-07	0.34 ± 0.03	0.30 ± 0.15	0.42 ± 0.03	0.39 ± 0.01	0.14	0.24
RIAN	−0.49 ± 0.03	−0.63 ± 0.04	−0.51 ± 0.01	−0.62 ± 0.03	−0.77 ± 0.03	**0.57**	1.08E-03	−0.77 ± 0.03	−0.39 ± 0.30	−0.61 ± 0.03	−0.64 ± 0.04	0.009	0.77
RAN_15_	n.d.	n.d.	n.d.	n.d.	n.d.	n.d.	n.d.	n.d.	n.d.	n.d.	n.d.	n.d.	n.d.
RAN_17_	162.0 ± 69.0	13.8 ± 8.0	103.7 ± 37.4	27.89 ± 13.98	24.38 ± 2.65	**0.51**	2.92E-03	24.38 ± 2.65	n.d.	n.d.	n.d.	n.d.	n.d.

Data are means of three biological replicates. Values after (±) are standard deviations. Correlations of the relative abundances of each 3-hydroxy fatty acid with either temperature or pH are provided with corresponding *p*-values. Significant correlations (*p* < 0.005) are indicated in bold. n.d.: not detected. Strain B: *Pedobacter lusitanus* NL19 (98.54% similarity).

**Table 6:** Relative percentage abundances of 3-hydroxy fatty acids vs. total 3-hydroxy fatty acids and related indices [defined by Wang et al. (2016)] for strain C cultivated under different temperature (at pH 5) and pH (at 25°C) conditions.

Strain C	Temperature conditions (°C)							pH conditions					
	5 °C	10°C	15°C	20°C	25°C	*R* ^2^	Value of *p*	5	6	7	8	*R* ^2^	Value of *p*
iso C_13_	n.d.	n.d.	n.d.	n.d.	n.d.	n.d.	n.d.	n.d.	n.d.	n.d.	n.d.	n.d.	n.d.
iso C_14_	0.31 ± 0.04	0.34 ± 0.14	0.16 ± 0.01	0.10 ± 0.09	0.03 ± 0.06	**0.69**	1.39E-04	0.03 ± 0.06	0.12 ± 0.06	0.23 ± 0.06	0.27 ± 0.04	**0.79**	1.24E-04
normal C_14_	0.79 ± 0.17	1.25 ± 0.54	1.32 ± 0.33	2.01 ± 0.26	2.48 ± 1.40	**0.50**	3.37E-03	2.48 ± 1.40	3.55 ± 1.29	3.11 ± 0.64	3.17 ± 0.62	0.04	0.54
iso C_15_	9.98 ± 1.84	16.57 ± 4.20	13.60 ± 0.82	24.36 ± 5.12	37.08 ± 9.96	**0.68**	1.41E-04	37.08 ± 9.96	36.44 ± 5.78	22.08 ± 2.60	26.93 ± 4.94	**0.37**	0.036
anteiso C_15_	23.04 ± 5.28	26.57 ± 5.97	17.95 ± 1.64	14.45 ± 3.63	6.45 ± 3.19	**0.67**	1.73E-04	6.45 ± 3.19	6.33 ± 1.00	4.55 ± 0.49	6.16 ± 1.26	0.03	0.58
normal C_15_	n.d.	n.d.	n.d.	n.d.	n.d.	n.d.	n.d.	n.d.	0.14 ± 0.25	0.13 ± 0.02	0.17 ± 0.07	0.19	0.15
iso C_16_	1.78 ± 0.23	1.54 ± 0.32	1.28 ± 0.13	1.40 ± 0.39	0.91 ± 0.05	**0.55**	1.47E-03	0.91 ± 0.05	1.02 ± 0.40	2.29 ± 0.18	1.79 ± 0.34	**0.51**	8.88E-03
normal C_16_	1.91 ± 0.48	1.54 ± 0.49	2.09 ± 0.30	3.77 ± 1.64	5.58 ± 1.78	**0.58**	9.16E-04	5.58 ± 1.78	5.03 ± 1.13	8.26 ± 0.52	7.54 ± 1.41	**0.36**	0.040
iso C_17_	12.65 ± 2.68	13.85 ± 2.78	19.64 ± 0.83	28.86 ± 2.92	34.01 ± 7.05	**0.83**	2.03E-06	34.01 ± 7.05	37.15 ± 7.24	48.26 ± 1.93	43.61 ± 3.99	**0.39**	0.029
anteiso C_17_	48.96 ± 3.95	38.19 ± 8.68	43.64 ± 1.73	24.11 ± 4.32	10.81 ± 5.00	**0.77**	1.61E-05	10.81 ± 5.00	9.65 ± 1.40	10.21 ± 1.93	9.25 ± 1.13	0.04	0.55
normal C_17_	0.50 ± 0.26	0.14 ± 0.02	0.19 ± 0.08	0.76 ± 1.07	2.41 ± 1.25	**0.37**	0.016	2.41 ± 1.25	0.30 ± 0.27	0.48 ± 0.26	0.71 ± 0.07	0.31	0.062
normal C_18_	0.07 ± 0.06	n.d.	0.12 ± 0.03	0.18 ± 0.03	0.24 ± 0.10	**0.59**	8.08E-04	0.24 ± 0.10	0.27 ± 0.03	0.40 ± 0.05	0.41 ± 0.09	**0.53**	7.21E-03
Total iso 3-OH FAs	24.71 ± 1.84	32.30 ± 2.07	34.69 ± 0.80	54.72 ± 3.29	72.03 ± 3.52	**0.90**	5.75E-08	72.03 ± 3.52	74.73 ± 1.25	72.85 ± 2.42	72.60 ± 0.89	7.99E-5	0.98
Total anteiso 3-OH FAs	72.00 ± 2.27	64.76 ± 3.05	61.59 ± 1.00	38.56 ± 0.71	17.26 ± 7.54	**0.88**	1.97E-07	17.26 ± 7.54	15.97 ± 0.50	14.76 ± 2.25	15.41 ± 0.18	0.05	0.48
Total normal 3-OH FAs	3.28 ± 0.57	2.94 ± 0.98	3.72 ± 0.22	6.72 ± 2.75	10.71 ± 4.18	**0.57**	1.12E-03	10.71 ± 4.18	9.30 ± 1.75	12.38 ± 0.18	11.99 ± 0.93	0.12	0.27
Iso/anteiso 3-OH FAs	0.34 ± 0.04	0.50 ± 0.06	0.56 ± 0.02	1.42 ± 0.11	5.09 ± 3.17	**0.48**	4.41E-03	5.09 ± 3.17	4.68 ± 0.07	5.03 ± 0.91	4.71 ± 0.08	4.23E-3	0.84
Iso/normal 3-OH FAs	7.63 ± 0.97	11.61 ± 2.82	9.33 ± 0.37	9.07 ± 3.40	7.26 ± 2.08	0.04	0.49	7.26 ± 2.08	8.26 ± 1.76	5.89 ± 0.28	6.08 ± 0.55	0.20	0.15
Anteiso/normal 3-OH FAs	22.46 ± 4.64	23.80 ± 7.98	16.59 ± 1.20	6.29 ± 2.04	1.92 ± 1.18	**0.78**	1.33E-05	1.92 ± 1.18	1.77 ± 0.40	1.19 ± 0.16	1.29 ± 0.11	0.21	0.14
RIAN	−1.47 ± 0.08	−1.53 ± 0.14	−1.41 ± 0.03	−1.16 ± 0.18	−0.94 ± 0.18	**0.67**	1.74E-04	−0.94 ± 0.18	−0.99 ± 0.09	−0.85 ± 0.007	−0.87 ± 0.04	0.16	0.19
RAN_15_	n.d.	n.d.	n.d.	n.d.	n.d.	n.d.	n.d.	n.d.	17.39	34.49 ± 7.10	40.13 ± 11.40	**0.45**	0.017
RAN_17_	126.5 ± 86.2	275.4 ± 91.9	258.3 ± 127.3	46.6 ± 45.6	6.27 ± 5.46	0.25	0.057	6.27 ± 5.46	22.98 ± 2.18	23.58 ± 7.50	13.10 ± 0.33	0.11	0.29

Data are means of three biological replicates. Values after (±) are standard deviations. Correlations of the relative abundances of each 3-hydroxy fatty acid with either temperature or pH are provided with corresponding *p*-values. Significant correlations (*p* < 0.005) are indicated in bold. n.d.: not detected. Strain C: *Pedobacter panaciterrae* (99.27% similarity).

The distribution of the individual 3-OH FAs was also observed to differ between the strains. In strain A, the *i*-C_15_ homologue was by far the most abundant 3-OH FA, followed by the *i*-C_17_ and *n*-C_16_ compounds, whatever the cultivation conditions (Figure 1; Table 4). In the case of strain B, the 3-OH FA distribution was dominated by either *i*-C_15_, *i*-C_17_ or *ai*-C_17_ homologues (depending on the cultivation conditions), followed by the *n*-C_16_ isomer (Table 4). As for strain C, the most abundant 3-OH FAs were *ai*-C_17_, *i-*C_17_, *ai*-C_15_ and *i*-C_15_, with a strong effect of the cultivation conditions on the relative proportion of these 3-OH FAs. It should be noted that part of the 3-OH FAs were present at very low abundance (<1% of total abundance in 3-OH FAs), and were even not detected in some of the samples. For example, the *i*-C_13_ and *n*-C_15_ homologues were mainly detected in strain A isolates (Table 4). In contrast, the *n*-C_18_ 3-OH FA was only detected in strains B and C (Tables 5, 6).

### 3.2. Influence of temperature on the FA distribution of the different strains

#### 3.2.1. Non-hydroxy fatty acids

The influence of temperature on the relative abundance of the individual FAs was compound- and strain-dependent. Regarding the non-hydroxy fatty acids, the relative abundance of *ai*-C_15:0_ and *n*-C_17:1_ was observed to significantly decrease with an increase in temperature for both strains A and B (Tables 1, 2). In addition to the two aforementioned homologues, temperature variations had a strong influence on the relative abundance of other FAs in strain A, as the relative abundances in *n*-C_15:0_ and *i*-C_15:0_ were shown to significantly increase with temperature (*R*^2^ = 0.94 and 0.54, respectively), whereas an opposite trend was observed for *i*-C_15:1_ (*R*^2^ = 0.95; Table 1). In contrast, temperature had only a limited impact on the non-hydroxy FA lipid profile in strain C (Table 3).

#### 3.2.2. 3-OH FAs

In all strains, the relative abundance of the total *anteiso* 3-OH FAs was observed to significantly decrease with increasing temperature (Tables 4–6). In addition, based on Spearman correlations, in strains A and C, the relative abundance of the total *normal* 3-OH FAs was significantly and positively correlated with temperature (*R*^2^ = 0.54 and 0.57, respectively; Tables 4, 6), whereas an opposite trend was observed for strain B (*R*^2^ = 0.57; Table 5). In strains B and C, the relative abundance of the total *iso* 3-OH FAs was also positively and strongly correlated with temperature (Tables 5, 6).

The relative abundance of some of the individual 3-OH FAs was also observed to be similarly affected by temperature changes, whatever the strain. Thus, in strain C, the relative abundance of the *ai*-C_17_ and *ai-*C_15_ 3-OH FAs significantly decreased with temperature (*R*^2^ = 0.77 and 0.67, respectively; Figure 1; Table 6). Such a significant decrease in the relative abundances of the *ai*-C_17_ and *ai*-C_15_ 3-OH FAs with temperature was similarly observed in strains B (*R*^2^ = 0.95 and 0.82, respectively; Table 5) and strain A (*R*^2^ = 0.79 and 0.85, respectively; Table 4) despite the low abundance of the two *anteiso* 3-OH FAs in the latter strain (< 5% of total 3-OH FAs).

In contrast, the effect of temperature differed between the strains for some of the individual 3-OH FAs. The relative abundance of the *i*-C_15_ 3-OH FA, predominant in strain A, did not change significantly with temperature (*R*^2^ = 0.02; Table 4), whereas it significantly increased for the B and C strains (*R*^2^ = 0.72 and 0.68, respectively; Tables 5, 6). Similarly, the relative abundance in the *i*-C_17_ 3-OH FA was observed to strongly (*R*^2^ = 0.86 and 0.83) and significantly increase with increasing temperature for strains B and C (Tables 5, 6), whereas no significant correlation was observed for strain A (Table 4). As for the relative abundance of the *i*-C_16_ homologue, it was observed to strongly and significantly decrease with temperature for strains A and B (Tables 4, 5), whereas only a weak change was noticed for strain C.

Regarding the *normal* 3-OH FAs, the relative abundance of the *n*-C_16_ homologue is significantly (*p* < 0.05) and positively correlated with temperature in strains A and C (*R*^2^ = 0.41 and 0.58, respectively; Tables 4, 6), while it is significantly and negatively correlated in strain B (*R*^2^ = 0.62; Table 5). As for the relative abundance of the *n*-C_15_ homologue, it is significantly and positively correlated with temperature in strain A (*R*^2^ = 0.73; *p* < 0.001), the only strain where it was detected.

### 3.3. Influence of pH on FA distribution in the different strains

Only weak to moderate significant correlations were observed between some of the individual non-hydroxy fatty acids and pH, with different trends from one strain to another (Tables 4–6). Similarly, the influence of pH on the relative abundance of 3-OH FAs was strain-dependent (Figure 2). In strain A, the relative abundances of the *i*-C_14_, *ai*-C_15_, *i*-C_16_, *i*-C_17_ and *ai*-C_17_ homologues were significantly and positively correlated with pH, whereas an opposite trend was observed for the *i*-C_13_ and *n*-C_15_ homologues (Table 4). No clear variations of the 3-OH FA relative abundance with pH were observed for strain B (Table 5). Finally, in strain C, a significant increase in the relative abundance of *i*-C_14_ and also *n*-C_18_ 3-OH FAs was observed with pH (Table 6).

### 3.4. Relationship between 3-OH FA-derived proxies and temperature/pH

**Figure 3:**
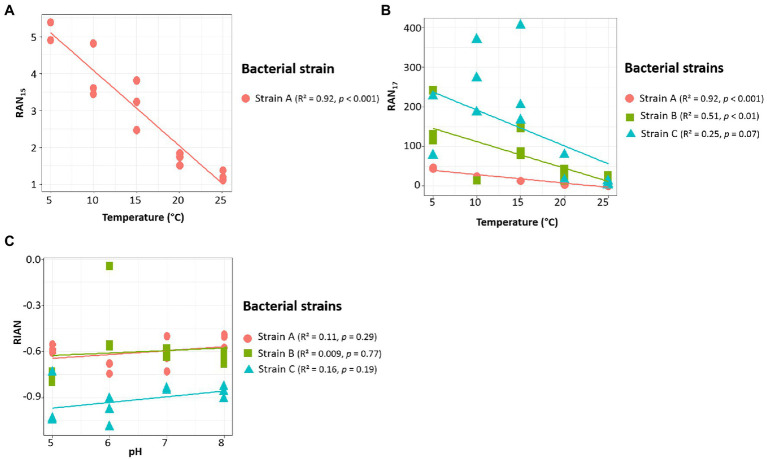
Relationships between temperature and RAN_15_ **(A)** and RAN_17_ **(B)** and between pH and RIAN **(C)** parameters (*cf.* 2.4 section for the equations of the indices) for the three Gram-negative bacterial strains isolated from soils of the French Alps. Strain A: *Flavobacterium pectinovorum* (98.78% similarity). Strain B: *Pedobacter lusitanus* NL19 (98.54% similarity). Strain C: *Pedobacter panaciterrae* (99.27% similarity). Each point corresponds to one replicate.

Wang et al. (2016) proposed three indices based on the relative abundance of 3-OH FAs and related to pH (RIAN, Eq. 1) or temperature (RAN_15_ and RAN_17_, Equations 2 and 3). The *n*-C_15_ homologue was not detected in strain B and in most of the cultures of strain C, preventing the calculation of the RAN_15_ for the corresponding samples of these two strains. In strain A, the RAN_15_ was observed to be significantly negatively correlated with temperature (*R*^2^ = 0.92; Table 4; Figure 3A). Regarding the RAN_17_, a significant negative relationship was observed between this index and the temperature for strains A (*R*^2^ = 0.93) and B (*R*^2^ = 0.51; Figure 3B; Tables 4–5). The RAN_17_ also tended to decrease with temperature for strain C (*R*^2^ = 0.25; Figure 3B; Table 6), even though this correlation was non-significant (*p* = 0.057). The RIAN (Eq. 1) did not show any correlation with pH for the three strains (Figure 3C; Tables 4–6).

## 4. Discussion

### 4.1. Choice of the investigated strains

Despite the lower relative abundance of *iso* and *anteiso* vs. *normal* homologues in most Gram-negative bacteria (e.g., Oyaizu and Komagata, 1983; Wilkinson, 1988) and consequently environmental samples, the straight chain, odd-carbon numbered 3-OH FAs are of special interest. They are at the basis of the different indices (RIAN, RAN_15_, and RAN_17_; Equations 1–3) based on the relative abundances of the 3-OH FAs and shown to be linearly correlated with soil pH and MAAT (Wang et al., 2016). Therefore, in this study, we purposefully selected, among 320 Gram-negative bacterial clones isolated from French Alps soils, three strains isolated from soils collected at different altitudes. These strains belong to the same phylum, *Bacteroidetes*, whose LPS was reported to be enriched in C_15_ and C_17_ 3-OH FAs (Wilkinson, 1988). Such a choice was made to ascertain the presence of the C_15_ and/or C_17_ 3-OH FAs in the investigated strains and to allow a proper investigation of the relationships between the aforementioned indices and temperature/soil pH.

The lipid profiles of the three strains were first determined at their optimal growth conditions (temperature of 25°C; pH = 5 for strain A and 8 for strains B and C). Regarding strain A, most closely related to *Flavobacterium pectinovorum,* the main non-hydroxy FAs were *n-*C_16:1,_ *i*-C_15_ and *n*-C_16:0_ homologues (Table 1), while the *i-*C_15_ and *i-*C_17_ compounds were the most abundant 3-OH FAs (Table 4). The lipid composition of strain A is consistent with the lipid profile reported by Cousin et al. (2007) for related strains *F. aquidurense* and *F. hercynium*. As for the strains B and C, most closely related to *Pedobacter lusitanus* and *Pedobacter panaciterrae,* respectively, their FA profiles were dominated by the *n*-C_16:1_ FA (Tables 2, 3) and the *i-*C_17_ and *i*-C_15_ 3-OH FAs (Tables 5, 6), in agreement with the FA distributions reported by Steyn et al. (1998), Yoon et al. (2007) and Covas et al. (2017) for related *Pedobacter* strains. Even though the predominant 3-OH FAs were, as expected, the same for the three investigated strains, the whole 3-OH FA distribution differed from one strain to another (Figures 1, 2). This is related to the fact that a large amount of phylogenetic and physiological diversity exists at the phylum or even at the genus level (Torsvik and Øvreås, 2002; Fierer et al., 2007).

### 4.2. Effect of growth temperature and pH on the FA lipid profile

#### 4.2.1. Effect of temperature

##### 4.2.1.1. Increase of the relative abundance of total *anteiso* FAs

Changes in growth temperature led to similar shifts in the FA lipid profile of the three investigated strains. Independently from the strain, an increase in the proportion of (i) the *ai-*C_15:0_ FA (Tables 1–3) and (ii) the total *anteiso* 3-OH FAs (Tables 4–6) with decreasing growth temperature was observed. Such a trend is consistent with the homeoviscous adaptative mechanism adopted by bacteria, since they are able to modulate the membrane lipid composition by producing lower-melting point fatty acids (unsaturated, short chain and branched chain fatty acids), which have a fluidizing effect on the membrane (Suutari and Laakso, 1994; Denich et al., 2003; Koga, 2012). As *anteiso* fatty acids have a lower melting point than *iso* and *normal* fatty acids (Russell and Fukunaga, 1990; Kaneda, 1991), an increase in their relative abundance is commonly observed in the bacterial cell membrane as growth temperature decreases (e.g., Oshima and Miyagawa, 1974; Mazzotta and Montville, 1997; Koga, 2012; Bajerski et al., 2017). Furthermore, the *anteiso*-positioned methyl groups cause greater fluidity of the membrane due to a greater disturbance of the packing order of the hydrocarbon chains (Siliakus et al., 2017). Thus, the increase in the relative abundance of all *anteiso* FAs – non-hydroxy but also 3-hydroxy – detected in the three *Bacteroidetes* strains of the present study fits with a common mechanism to maintain the cell membrane fluidity at low temperatures.

##### 4.2.1.2. Total *anteiso* 3-OH FAs versus total *iso* and *normal* 3-OH FAs

A decrease in the relative proportion of *iso* FAs at lower temperatures is often concomitant with higher relative abundances in *anteiso* FAs (e.g., Kaneda, 1991; Prakash et al., 2015). For example, a higher relative abundance of lower-melting point *anteiso* FAs was observed at lower temperature for the Gram-positive species *Bacillus subtilis* and *Bacillus megaterium*, together with a lower relative abundance of *iso* FAs (Suutari and Laakso, 1992). Similarly, the proportion of the *i*-C_15_ and -C_17_ FAs of ten Gram-negative *Thermus* strains decreased and concomitantly the *ai*-C_15_ and -C_17_ FA increased with decreasing temperature (Nordström and Laakso, 1992). Here, the ratio of the total *iso* 3-OH FAs versus total *anteiso* 3-OH FAs was observed to increase with increasing temperature independently from the strain (Tables 4–6), confirming the importance of the branching pattern in response to changing temperature.

This conclusion is strengthened by a concomitant decrease in the total *anteiso* 3-OH FAs vs. total *normal* 3-OH FAs with increasing temperature observed for the three investigated strains (Tables 4–6). A similar trend was recently observed in Gram-negative and Gram-positive psychrophilic bacteria isolated from Pakistan glaciers, for which a higher temperature resulted in a significant increase in the relative abundance of saturated *normal* FAs and a substantial decrease in the relative abundance of branched FAs (Hassan et al., 2020). The change of the 3-OH FA branching ratio with temperature, reflected by the relative increase and decrease of the ratios in *anteiso*/*normal* and *iso*/*anteiso* 3-OH FAs at lower temperature, respectively, should enable the membrane to maintain a normal liquid-crystalline state and the cell to function normally even at low temperature, as previously suggested for non-hydroxy fatty acids (Suutari and Laakso, 1994; Sun et al., 2012). This appears as a common adaptation mechanism to temperature for all the *Bacteroidetes* strains investigated in this study.

##### 4.2.1.3. Strain-specific trends

Despite shared variations in the ratios of the main 3-OH FAs with temperature, strain-specific trends were also observed when examining the individual relative abundances in 3-OH FAs. Thus, the variations of the relative abundance of the *iso* 3-OH FAs with temperature generally differed between strain A and the two other strains, the total relative abundance in *iso* 3-OH FAs being only weakly correlated with temperature in strain A (Table 4), in contrast with strains B and C (Tables 5, 6). This might be related to the fact that strain A is related to the genus *Flavobacterium* (class *Flavobacteria*) whereas the genus of strains B and C is *Pedobacter* (class *Sphingobacteria*; Hahnke et al., 2016). Strain-specific trends were also observed for the *normal* 3-OH FAs, despite their lower abundance than that of the *iso* and *anteiso* isomers. However, the relative abundance of the *normal* 3-OH FAs (C_14_-C_18_) significantly increased with temperature, especially at 20 and 25°C, for strains A and C (Tables 4, 6), whereas no correlation was observed for strain B (Table 5). This implies that the relatedness to a different genus cannot alone explain the observed trends, suggesting that even within a genus two strains could respond differently to environmental changes (e.g., strains B and C). Altitude was shown to affect physicochemical characteristics and microbial diversity in soils (Zhang et al., 2022). Therefore, as the strains investigated in the present study were isolated from three soils collected at different altitudes in the French Alps, it cannot be excluded that the specific variations in 3-OH FA relative abundances may at least partly reflect an intrinsic adaptation of each bacterial strain to the habitat it was isolated from. This hypothesis could explain the differences in the values of the ratios in *anteiso*/*normal* and *iso*/*anteiso* 3-OH FAs between the three strains at a given temperature. The applicability of 3-OH FAs as temperature proxies in soils could therefore be strongly influenced by the Gram-negative bacterial diversity, as previously suggested (e.g., Huguet et al., 2019; Véquaud et al., 2021b).

In addition to the branched FAs, the role of FA unsaturation in cold adaptation has previously been reported, with higher relative abundances of monounsaturated FAs observed at lower temperatures in several Gram-negative bacterial species (e.g., *Rhizobium leguminosarum,* Théberge et al., 1996; *Chryseobacterium frigidisoli*, Bajerski et al., 2017). Such a trend was explained by the lower melting point of monounsaturated FAs vs. saturated counterparts (Russell, 1984, 1989) and is consistent with the increase in the relative abundance of (i) the *n*-C_17:1_ FA observed in strain B and to a lower extent in strain A (Tables 1, 2) and (ii) *i*-C_15:1_ FA observed in strain A (Table 1) at lower temperatures. This is also in line with the increase in the relative abundance of the total unsaturated FAs with decreasing temperature observed in strain B (Table 2).

Overall, even though 3-OH FAs are generally less abundant than non-hydroxy FAs in the cell membrane of the investigated bacterial species, it is the first time to our knowledge that structural changes in 3-OH FAs in response to temperature variations are described in *Bacteroidetes* soil species. A notable decrease in the ratio of *iso* vs. *anteiso* 3-OH FAs with decreasing temperature was observed for all investigated strains. Such a shared temperature adaptation mechanism should be further confirmed by investigating additional Gram-negative bacterial strains from different settings. The relative proportions of the individual 3-OH FAs were affected by temperature variations, but the corresponding trends were strain-dependent. This could influence the general applicability of 3-OH FAs as temperature proxies in soils.

#### 4.2.2. Effect of pH

In contrast with temperature, less data is available on the lipid membrane adaptation to pH variations, with contrasting observations, based on a limited number of groups of organisms. Prakash et al. (2015) observed an increase in the relative proportion of branched-chain FAs (*i*-C_15_, *i*-C_16_, *ai*-C_15_, *ai*-C_17_) at higher pH for the Gram-positive *Micrococcus yunnanensis* and *Micrococcus aloeverae*. Giotis et al. (2007) similarly observed that *iso* and *anteiso* FAs where important for pH adaptation of the Gram-negative *Listeria Monocytogenes*, with an increase in the relative abundance of branched-chain FAs in alkaline conditions and the opposite trend under acidic conditions. An increase in the relative proportion of short-chain *i*- and *ai*-C_15:0_ FAs at pH 8 and a decrease at lower pH (<6) was also observed for *Chryseobacterium frigisidoli*, isolated from Antarctic glacier soils (Bajerski et al., 2017). In contrast, Russell (1984) reported an increase of *iso* and *anteiso* FAs at low pH, and Bååth and Anderson (2003) a decrease of *i*-C_15:0_ and *i*-C_16:0_ at higher pH, with no obvious change in the relative abundance of *anteiso* FAs.

The interpretation of the fatty acid variations in response to changing pH is therefore complex. Different trends were observed between the three *Bacteroidetes* strains investigated. In strain B, pH did not have a major effect on the FA composition. In strains A and C, the relative abundance of *i*-C_17_ 3-OH FA was observed to increase at higher pH (Tables 4, 6). Nevertheless, in these two strains, the relative abundance of *i*-C_15_ 3-OH FA, the most abundant of the 3-OH FAs, decreased with increasing pH (Tables 4, 6), thus showing an opposite trend to *i*-C_17_ 3-OH FA. In parallel, the relative abundances of *ai*-C_15_ and C_17_ 3-OH FAs were observed to increase at higher pH for strain A (Table 4). Regardless of the increasing or decreasing variations in the relative abundances of the *iso* and *anteiso* isomers with pH, the ratio of the *iso*/*anteiso* 3-OH FAs was shown to be significantly and negatively correlated with pH in strain A, concomitantly with a significant increase of the *anteiso/normal* 3-OH FA ratio (Table 4). The relative increase in branched *anteiso* 3-OH FAs could help in maintaining the membrane flexibility at higher pH (Russell, 1989), as was also assumed for *C. frigisidoli* (Bajerski et al., 2017). Nevertheless, why such a change would only occur at alkaline pH and only for strain A is unclear. Further studies are needed to better understand the structural membrane adaptation of Gram-negative bacteria in response to pH variations at the level of 3-OH FAs.

### 4.3. Implications for the use of 3-OH FAs as temperature and pH proxies

3-OH FAs were recognized as potential temperature and pH proxies in soils 6 years ago after their analysis in 26 Chinese soils (Wang et al., 2016). When considering the different indices proposed by these authors, the RAN_17_ calculated using the data obtained from each of the three *Bacteroidetes* strains appears to be weakly to strongly correlated with the growth temperature (Figure 3B; Tables 4–6). The RAN_17_/temperature relationships present different slopes and y-intercept. This can explain, at least partly, the difficulty in establishing global RAN_17_/MAAT calibrations from soils distributed worldwide, since the bacterial diversity is expected to widely vary between soils, especially for 3-OH FA-producing Gram-negative microorganisms (e.g., Margesin et al., 2009; Siles and Margesin, 2016). Our study, based on data obtained at the bacterial cell level, confirms the potential of the RAN_17_ as a temperature proxy in terrestrial settings at a regional scale (Véquaud et al., 2021a; Wang et al., 2021).

The global application of the RAN_15_ as a temperature proxy appears even more challenging since the 3-OH FAs required for the calculation of this index can only be detected in a subset of Gram-negative bacterial strains. In this study the only strain for which the RAN_15_ could be calculated was strain A, and a strong correlation with MAAT was obtained (Figure 3A; Table 4). This implies that the application of the RAN_15_ as a temperature proxy highly depends on whether the soil Gram-negative bacterial community produces *ai*- and *n*-C_15_ 3OH FAs in sufficient amounts.

Regarding the RIAN index, no significant correlations with pH were observed for the three investigated strains (Figure 3C; Tables 4–6). In soils, the weakness of the correlations between the RIAN and pH were notably explained by the fact that (i) MAAT rather than soil pH may instead influence bacterial diversity and composition and by (ii) the intrinsic heterogeneity of soils, with a large diversity of bacterial communities encountered at a given site (Véquaud et al., 2021a). The first hypothesis is consistent with the fact that the RIAN index was significantly and moderately correlated with MAAT for the three strains (*R*^2^ = 0.51–0.67; Tables 4–6), reflecting temperature having a stronger influence over pH on this ratio. In addition, the response of Gram-negative bacterial communities to pH was shown to be complex, making the use of the RIAN as a pH proxy in soils difficult, as the microbial diversity naturally varies from one sample to another (Margesin et al., 2009; Siles and Margesin, 2016).

Non-parametric machine learning models were recently used (Véquaud et al., 2021a; Wang et al., 2021) to take into account non-linear environmental influences, as well as the intrinsic complexity of environmental settings. Véquaud et al. (2021a) especially used a random forest algorithm, which allowed the development of strong global calibrations with MAAT/pH. The MAAT random forest model included all 3-OH FAs involved in the calculation of the RAN_15_ and RAN_17_ indices (*ai*-C_15_ and -C_17_ and *n*-C_15_ and -C_17_), with other individual 3-OH FAs also having a major weight (e.g., *i*-C_13_, *i*-C_14_, *n*-C_16_; Véquaud et al., 2021a). This is consistent with the results we obtained at the microbial level, where (i) the adaptation mechanism of the *Bacteroidetes* strains to temperature variations involved a concomitant change in the ratios of the *iso*, *anteiso* and *normal* 3-OH FAs and (ii) the growth temperature was observed to have a significant effect on the relative abundance of most of the individual 3-OH FAs (Tables 4–6). As the whole suite of 3-OH FAs can be influenced by temperature, our results highlight the fact that the global models based on these compounds should preferentially include all of them rather than a limited set as in the RAN_15_ and RAN_17_ indices, in order to reflect the natural response of Gram-negative bacteria to temperature changes.

A similar conclusion can be drawn for the global calibrations between pH and 3-OH FA distribution. Even though in the random forest pH model proposed by Véquaud et al. (2021a) *i*-C_13_, *i*-C_16_ and *n*-C_15_ 3-OH FAs were shown to have a major weight, all the C_10_ to C_18_ 3-OH FAs had a non-negligible influence. This is in agreement with the results derived from the three *Bacteroidetes* species investigated in the present study, as pH was shown to have a potential influence on the relative distribution of all the individual 3-OH FAs (Tables 4–6), despite different variations from one strain to another.

The present study focused on three *Bacteroidetes* strains isolated from different soils, each presenting a specific lipid profile (Figures 1, 2). Nevertheless, soils are naturally composed of a large diversity of Gram-negative bacteria (Margesin et al., 2009) which all produce 3-OH FAs and where *Bacteroidetes* might not be predominant. Hence, additional strains of Gram-negative bacteria should be investigated to better understand the lipid adaptation mechanism of these microorganisms to temperature and pH changes and to better constrain the applicability of 3-OH FAs as environmental proxies in terrestrial settings.

## 5. Conclusion

3-OH FAs were recently proposed as promising temperature and pH (paleo)proxies in soil. Nevertheless, to date, the relationships between the 3-OH FA distribution and temperature/pH have only been based on empirical studies in soil, with no work performed at the microbial level. In this work, the influence of growth temperature and pH on the lipid profile of three different strains of soil Gram-negative bacteria, belonging to the same phylum (*Bacteroidetes*) but isolated from different altitudinal habitats, was investigated. Even though the non-hydroxy FAs were more abundant than the 3-OH FAs in the three investigated strains, we showed that 3-OH FAs are involved in the membrane adaptation of *Bacteroidetes* to temperature. The three *Bacteroidetes* strains under study shared a common temperature adaptation mechanism, with a significant decrease in the ratio of *iso* versus *anteiso* or *normal* 3-OH FAs at lower temperature. Nevertheless, the variations of the relative abundances of the individual 3-OH FAs with temperature were shown to be strain-dependent. This might be due to the fact that the three investigated strains are different species, but might also be related to the environment from which they were originally isolated, characterized by contrasting temperature regimes. In contrast to the growth temperature, no common adaptation mechanism to pH was noticed, the variations of the FA lipid profiles differing between species. As the entire suite of 3-OH FAs present in the lipid membrane of *Bacteroidetes* can be influenced by either temperature or pH, our results suggest that models based on these compounds and envisioning the reconstruction of environmental changes in terrestrial settings at the global scale should include all compounds rather than defining indices based on a sub-selection as initially proposed. In a context of global change, additional studies on a larger number of Gram-negative bacterial strains from contrasting settings (soils, marine and lake environments) are now required to better understand the lipid adaptation mechanism of these microorganisms to environmental variations.

## Data availability statement

The datasets presented in this study can be found in online repositories. The names of the repository/repositories and accession number(s) can be found in the article/Supplementary material.

## Author contributions

EH, SC, and AH designed the research work. PV collected the soil samples from the French Alps. MS-C and SC isolated and identified bacterial strains. EH carried out the cultivation of the strains, lipid extraction and analysis. CA carried out the GC/MS analysis. EH and AH carried out the statistical analyses and generated the figures and tables. AH and SC supervised the whole research work. EH, AH, and SC wrote the original draft. AK provided critical reading and assisted in the writing and revision of the manuscript. All authors contributed to the article and approved the submitted version.

## Funding

Funds for the research work were provided by Sorbonne Université through the TEMPO project (Emergence 2019-2020). In addition, the EC2CO program (CNRS/INSU – BIOHEFECT/MICROBIEN) also supported this study through the SHAPE project.

## Conflict of interest

The authors declare that the research was conducted in the absence of any commercial or financial relationships that could be construed as a potential conflict of interest.

## Publisher’s note

All claims expressed in this article are solely those of the authors and do not necessarily represent those of their affiliated organizations, or those of the publisher, the editors and the reviewers. Any product that may be evaluated in this article, or claim that may be made by its manufacturer, is not guaranteed or endorsed by the publisher.
